# Stage-driven mortality and clinical risk stratification among lung cancer patients in Dhaka, Bangladesh: A multi-hospital secondary analysis

**DOI:** 10.1371/journal.pone.0359406

**Published:** 2026-10-15

**Authors:** Sourov Das, Sushmita Kundu, Salma Jahan Tanny

**Affiliations:** 1 Comilla Medical College, Cumilla, Bangladesh; 2 Tbilisi State Medical University, Tbilisi, Georgia; KPC Medical College and Hospital, INDIA

## Abstract

**Introduction:**

Lung cancer is a major cause of cancer mortality, and patient-level outcome data from Bangladesh remain limited. Locally relevant, interpretable risk-stratification models may help identify patients with higher recorded mortality risk using routinely available clinical variables. This study evaluated mortality-associated factors and the hospital-wise internal-external validation performance of a clinical risk-stratification model among lung cancer patients in Dhaka, Bangladesh.

**Methods:**

We performed a retrospective secondary analysis of a publicly available, de-identified multi-hospital dataset comprising 1,782 patients. The primary endpoint was recorded binary mortality status, defined from the source variable survived (No = mortality event; Yes = no recorded mortality event). Multivariable logistic regression was used for association analysis. A hospital-agnostic logistic prediction model was evaluated using leave-one-hospital-out internal-external validation. Discrimination was assessed by the area under the receiver operating characteristic curve (AUROC), calibration by Brier score, calibration intercept and slope, and predicted-risk deciles, and potential clinical utility by decision curve analysis.

**Results:**

Among 1,782 patients, 770 (43.2%) were recorded as survived = No. Recorded mortality increased across cancer stages: 16.3% in Stage I, 32.5% in Stage II, 55.6% in Stage III, and 79.2% in Stage IV. Compared with Stage I, adjusted odds ratios were 2.54 (95% CI 1.86 to 3.46) for Stage II, 6.66 (95% CI 4.86 to 9.14) for Stage III, and 20.77 (95% CI 14.46 to 29.83) for Stage IV. Surgery was associated with lower adjusted odds than chemotherapy (adjusted OR 0.65, 95% CI 0.48 to 0.89). Apparent AUROC was 0.769; pooled leave-one-hospital-out AUROC was 0.753 with a Brier score of 0.198. Pooled calibration yielded an intercept of −0.031 and slope of 0.911. At the evaluated 0.05 increments, the model had greater net benefit than treat-all and treat-none strategies from approximately 0.20 to 0.75.

**Conclusion:**

Recorded mortality in this multi-hospital Bangladeshi lung cancer dataset was strongly stage-driven. An interpretable logistic risk model showed moderate discrimination and reasonable calibration across hospital-wise internal-external validation. These findings support further evaluation of routinely available clinical risk stratification, but independent external and prospective validation are required before clinical implementation.

## Introduction

### Global and Bangladesh lung cancer burden

Lung cancer remains one of the leading causes of cancer mortality worldwide. GLOBOCAN 2022 estimated approximately 2.48 million new lung cancer cases and 1.82 million lung cancer deaths globally, making lung cancer the leading cause of cancer death [1]. In Bangladesh, GLOBOCAN estimates indicate a substantial national cancer burden, with lung cancer contributing importantly to cancer morbidity and mortality [2].

Despite this burden, patient-level lung cancer outcome data from Bangladesh remain limited. Cancer outcomes in low- and middle-income countries may be influenced by delayed presentation, limited diagnostic access, constrained treatment infrastructure, and barriers within care pathways [3–6].

### Tobacco, air pollution, and delayed diagnosis context

Tobacco exposure remains an important public-health issue in Bangladesh [7]. Environmental exposure is also relevant because Bangladesh has high ambient PM2.5 air pollution, and outdoor air pollution is an established carcinogenic exposure [8–10]. These population-level exposures provide context for lung cancer burden, although detailed quantitative exposure measures were not available in the present dataset.

Delayed diagnosis and treatment access are additional challenges in low-resource settings. Limited specialist oncology capacity, diagnostic delays, and referral barriers may contribute to advanced-stage presentation and worse outcomes [3–6].

### Need for locally relevant risk stratification

Clinical risk stratification may help identify patients at higher recorded mortality risk and support treatment planning, referral decisions, clinical review prioritization, and health-system planning. In resource-limited settings, interpretable models based on routinely available clinical variables may be more feasible than approaches requiring detailed molecular, imaging, or genomic data.

A multi-hospital dataset also permits assessment of whether model performance is reasonably stable across hospital sources rather than only within a single center. The objective of this study was therefore to evaluate demographic, clinical, lifestyle, comorbidity, stage, and treatment-related factors associated with recorded mortality and to assess the discrimination, calibration, potential clinical utility, and hospital-wise transportability of an interpretable logistic risk model.

## Methods

### Study design and data source

This study was a retrospective secondary analysis of the publicly available, de-identified dataset “A Multi-Hospital Clinical Dataset for Lung Cancer Mortality and Survival Analysis in Dhaka, Bangladesh,” obtained from Mendeley Data (https://doi.org/10.17632/wzzxjs8gww.1) [11]. The dataset was accessed on 2 February 2026 and contains demographic, hospital-source, clinical, lifestyle, comorbidity, treatment, date, and recorded outcome variables for 1,782 patients with lung cancer. The authors had access only to the publicly released de-identified dataset and had no access to information capable of identifying individual participants.

### Study setting and population

The dataset included records attributed to four hospitals and medical centers in Dhaka, Bangladesh: National Institute of Cancer Research & Hospital, United Hospital Limited, Square Hospital Ltd., and Evercare Hospital Dhaka.

All 1,782 records available in the public dataset were included in the analysis. During the initial data-quality assessment, missingness was evaluated for each variable, and duplicate patient identifiers and duplicate complete records were assessed. No missing values were present in any variable in the publicly released dataset, and no duplicate patient identifiers or duplicate full records were detected. Consequently, no records were excluded because of missing data and no imputation was performed. Because this was a secondary analysis of a publicly available dataset, we could not determine whether any missing-data handling, data cleaning, or record exclusion had occurred before the dataset was released publicly.

### Variables and outcome definition

The dataset included age, gender, hospital source, cancer stage, family history of cancer, smoking status, body mass index, cholesterol level, hypertension, asthma, cirrhosis, history of other cancer, treatment type, diagnosis date, end-treatment date, and the source outcome variable survived. The primary endpoint for this analysis was recorded mortality status: records with survived = No were coded as mortality = 1, and records with survived = Yes were coded as mortality = 0.

The source dataset did not provide a verified date of death or a censoring time suitable for overall-survival analysis. The diagnosis and end-treatment dates were therefore not interpreted as event-time and censoring data and were not used for Kaplan-Meier, Cox proportional-hazards, or other time-to-event analyses. Accordingly, the reported odds ratios and predicted probabilities describe the recorded binary mortality endpoint and should not be interpreted as hazard ratios, median overall survival, or time-specific survival probabilities.

For descriptive analyses, Stage III and Stage IV were grouped as advanced-stage disease and Stage I and Stage II as early-stage disease. Body mass index was categorized as underweight (<18.5 kg/m2), normal/low (18.5 to 22.9 kg/m2), overweight (23.0 to 27.4 kg/m2), and obese (>=27.5 kg/m2).

### Statistical analysis

Continuous variables were summarized using medians and interquartile ranges, and categorical variables using frequencies and percentages. Characteristics were compared between records with survived = Yes and survived = No using Mann-Whitney U tests for continuous variables and chi-square tests for categorical variables. Reporting of the observational component was guided by STROBE principles [12].

Univariable logistic regression was used to estimate crude odds ratios for recorded mortality. The multivariable association model included age, gender, hospital source, cancer stage, family history, smoking status, body mass index, cholesterol level, hypertension, asthma, cirrhosis, other-cancer history, and treatment type, and reported adjusted odds ratios with 95% confidence intervals. These 13 predictor variables corresponded to 21 estimated non-intercept coefficients after categorical expansion. With 770 mortality events, the association model had 36.7 events per estimated predictor parameter. Multicollinearity was assessed using variance inflation factors. For the continuous predictors age, body mass index, and cholesterol level, potential nonlinearity was assessed by adding quadratic terms jointly and comparing model fit using a likelihood-ratio test.

### Prediction model development and internal-external validation

For prediction, a separate hospital-agnostic multivariable logistic regression model was specified using the same clinical predictors except hospital source. Hospital source was excluded a priori because hospital identity cannot function as a transportable predictor when an entire hospital is held out for validation. The prediction model contained 18 non-intercept coefficients, corresponding to 42.8 events per estimated predictor parameter. Predictors were prespecified from variables available in the source dataset; no automated feature selection, stepwise selection, hyperparameter optimization, or data-driven probability-threshold optimization was performed. Logistic regression was selected for interpretability and to provide predicted probabilities alongside clinically interpretable coefficients. Prediction-model reporting was guided by TRIPOD, TRIPOD+AI, and TRIPOD-Cluster recommendations [13–16]. Full regression coefficients for the association and prediction models are provided in S2 Table.

Overall performance of the hospital-agnostic prediction model in the full cohort was reported as apparent performance. To assess hospital-wise transportability and reduce reliance on apparent performance, leave-one-hospital-out internal-external validation was performed. In each of four iterations, one hospital was held out, the unpenalized logistic regression model was refitted on the other three hospitals, and predictions were generated for the held-out hospital. Predictions from all four held-out hospitals were then pooled so that every patient had a prediction generated from a model that had not been fitted using that patient or that hospital.

Discrimination was assessed using AUROC. The Brier score quantified overall prediction error. Classification metrics at a probability threshold of 0.5 were reported descriptively; the 0.5 threshold was not optimized. Calibration of the pooled out-of-hospital predictions was assessed using calibration intercept, calibration slope, and observed versus predicted mortality across predicted-risk deciles [17,18]. Decision curve analysis compared the model with treat-all and treat-none strategies at thresholds from 0.05 to 0.80 in increments of 0.05 [19–21].

All analyses were performed in Python. The reproducible workflow included data-quality checks, descriptive analyses, logistic regression, model diagnostics, leave-one-hospital-out internal-external validation, calibration assessment, decision curve analysis, and generation of tables and figures. The analysis code used for the revised results is provided as S1 Code.

### Ethics statement

This secondary analysis used only a publicly available, fully de-identified dataset and involved no participant contact or access to personally identifiable information. On this basis, the authors determined that additional institutional ethics approval was not required for this secondary analysis. The authors did not obtain informed consent for this secondary analysis because only publicly available, de-identified data were analyzed. The source dataset states that the records were anonymized and intended for academic research use.

**Artificial intelligence tools:** OpenAI ChatGPT was used solely for language and grammatical editing. All AI-assisted content was subsequently reviewed and verified by the authors, who retain full responsibility for the accuracy, integrity, and final content of the manuscript.

## Results

### Study cohort and baseline characteristics

A total of 1,782 records were analyzed. Of these, 770 were recorded as survived = No, corresponding to a recorded mortality proportion of 43.2%, while 1,012 were recorded as survived = Yes. NICRH contributed 470 records, United Hospital Limited 443, Square Hospital Ltd. 436, and Evercare Hospital Dhaka 433. Baseline characteristics by recorded mortality status are summarized in Table 1.

**Table 1:** Baseline characteristics of lung cancer patients by recorded mortality status.

Characteristic	Recorded as survived = Yes	Recorded as survived = No	Total	P value
Age, median (IQR)	57.0 (41.0 to 74.0)	56.0 (39.0 to 71.0)	57.0 (40.0 to 73.0)	0.1725
**Gender**				0.1878
Male	494 (48.8%)	401 (52.1%)	895 (50.2%)	
Female	518 (51.2%)	369 (47.9%)	887 (49.8%)	
**Hospital**				0.9923
NICRH	266 (26.3%)	204 (26.5%)	470 (26.4%)	
United Hospital Limited	252 (24.9%)	191 (24.8%)	443 (24.9%)	
Square Hospital Ltd.	250 (24.7%)	186 (24.2%)	436 (24.5%)	
Evercare Hospital Dhaka	244 (24.1%)	189 (24.5%)	433 (24.3%)	
**Cancer stage**				<0.001
Stage I	384 (37.9%)	75 (9.7%)	459 (25.8%)	
Stage II	364 (36.0%)	175 (22.7%)	539 (30.2%)	
Stage III	190 (18.8%)	238 (30.9%)	428 (24.0%)	
Stage IV	74 (7.3%)	282 (36.6%)	356 (20.0%)	
**Advanced stage**				<0.001
Stage I/II	748 (73.9%)	250 (32.5%)	998 (56.0%)	
Stage III/IV	264 (26.1%)	520 (67.5%)	784 (44.0%)	
**Family history**				0.8117
No	652 (64.4%)	491 (63.8%)	1143 (64.1%)	
Yes	360 (35.6%)	279 (36.2%)	639 (35.9%)	
**Smoking status**				0.7621
Current Smoker	376 (37.2%)	272 (35.3%)	648 (36.4%)	
Never Smoked	276 (27.3%)	223 (29.0%)	499 (28.0%)	
Former Smoker	274 (27.1%)	204 (26.5%)	478 (26.8%)	
Passive Smoker	86 (8.5%)	71 (9.2%)	157 (8.8%)	
BMI, median (IQR)	25.4 (20.9 to 30.1)	25.7 (21.0 to 30.1)	25.6 (20.9 to 30.1)	0.5916
**BMI group**				0.9381
Obese	397 (39.2%)	299 (38.8%)	696 (39.1%)	
Overweight	251 (24.8%)	201 (26.1%)	452 (25.4%)	
Normal/low	236 (23.3%)	175 (22.7%)	411 (23.1%)	
Underweight	128 (12.6%)	95 (12.3%)	223 (12.5%)	
Cholesterol level, median (IQR)	226.0 (180.0 to 276.0)	229.0 (184.0 to 272.0)	226.5 (181.0 to 275.0)	0.9854
**Hypertension**				0.4025
No	602 (59.5%)	474 (61.6%)	1076 (60.4%)	
Yes	410 (40.5%)	296 (38.4%)	706 (39.6%)	
**Asthma**				0.7332
No	764 (75.5%)	575 (74.7%)	1339 (75.1%)	
Yes	248 (24.5%)	195 (25.3%)	443 (24.9%)	
**Cirrhosis**				0.7320
No	914 (90.3%)	700 (90.9%)	1614 (90.6%)	
Yes	98 (9.7%)	70 (9.1%)	168 (9.4%)	
**Other cancer**				0.6614
No	844 (83.4%)	649 (84.3%)	1493 (83.8%)	
Yes	168 (16.6%)	121 (15.7%)	289 (16.2%)	
**Treatment type**				0.1948
Radiation	263 (26.0%)	210 (27.3%)	473 (26.5%)	
Combined	251 (24.8%)	196 (25.5%)	447 (25.1%)	
Chemotherapy	235 (23.2%)	197 (25.6%)	432 (24.2%)	
Surgery	263 (26.0%)	167 (21.7%)	430 (24.1%)	

Values are n (%) unless otherwise stated. Percentages in the two outcome columns use column totals as denominators. P values are from chi-square tests for categorical variables and Mann-Whitney U tests for continuous variables. BMI, body mass index; IQR, interquartile range.

### Stage-specific and treatment-specific recorded mortality

Recorded mortality increased progressively with advancing cancer stage: 16.3% in Stage I, 32.5% in Stage II, 55.6% in Stage III, and 79.2% in Stage IV. When grouped, recorded mortality was 25.1% for Stage I/II and 66.3% for Stage III/IV disease (Fig 1).

**Fig 1:**
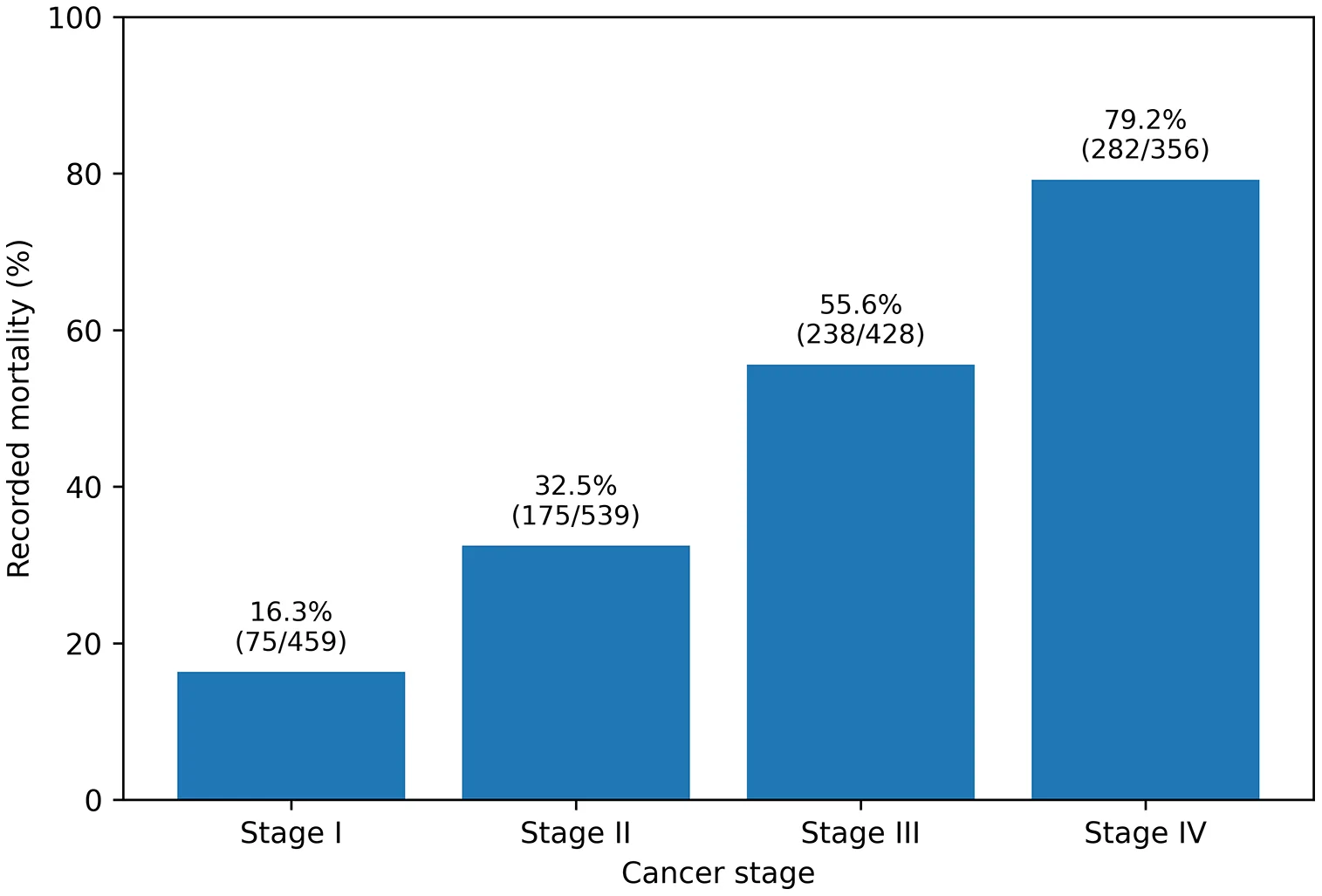
Stage-wise recorded mortality among lung cancer patients. The bar chart shows the proportion recorded as survived = No within each cancer stage, with event counts and denominators displayed above the bars.

Crude recorded mortality was 45.6% among patients receiving chemotherapy, 43.8% for combined treatment, 44.4% for radiation, and 38.8% for surgery. These crude treatment comparisons should not be interpreted causally because treatment selection is related to stage, fitness, resectability, intent, and other clinical factors not fully captured in the source dataset.

### Factors associated with recorded mortality

In multivariable analysis, cancer stage showed the strongest adjusted association with recorded mortality. Compared with Stage I, the adjusted odds ratio was 2.54 (95% CI 1.86 to 3.46) for Stage II, 6.66 (95% CI 4.86 to 9.14) for Stage III, and 20.77 (95% CI 14.46 to 29.83) for Stage IV. Surgery was associated with lower adjusted odds than chemotherapy (adjusted OR 0.65, 95% CI 0.48 to 0.89; p = 0.0067). Hospital source was not significantly associated with recorded mortality after adjustment. Full univariable and multivariable results are shown in Table 2.

**Table 2:** Univariable and multivariable logistic regression for recorded mortality.

Predictor	Univariable OR (95% CI)	Univariable P value	Multivariable aOR (95% CI)	Multivariable P value
Age	0.997 (0.992 to 1.001)	0.1685	0.997 (0.992 to 1.003)	0.3269
Male vs female	1.140 (0.945 to 1.375)	0.1723	1.059 (0.855 to 1.311)	0.6007
NICRH vs Evercare	0.990 (0.761 to 1.288)	0.9409	1.037 (0.769 to 1.398)	0.8109
Square vs Evercare	0.961 (0.734 to 1.256)	0.7687	1.073 (0.790 to 1.456)	0.6524
United vs Evercare	0.979 (0.749 to 1.278)	0.8734	1.062 (0.783 to 1.439)	0.7000
Stage II vs Stage I	2.462 (1.812 to 3.343)	<0.001	2.538 (1.863 to 3.459)	<0.001
Stage III vs Stage I	6.413 (4.693 to 8.765)	<0.001	6.661 (4.855 to 9.139)	<0.001
Stage IV vs Stage I	19.511 (13.667 to 27.855)	<0.001	20.769 (14.462 to 29.826)	<0.001
Family history yes vs no	1.029 (0.847 to 1.251)	0.7733	1.153 (0.923 to 1.442)	0.2099
Former smoker vs current smoker	1.029 (0.810 to 1.307)	0.8135	0.941 (0.716 to 1.235)	0.6604
Never smoked vs current smoker	1.117 (0.883 to 1.414)	0.3576	1.151 (0.881 to 1.504)	0.3030
Passive smoker vs current smoker	1.141 (0.804 to 1.621)	0.4605	1.194 (0.803 to 1.776)	0.3806
BMI	1.005 (0.987 to 1.022)	0.6089	1.009 (0.989 to 1.029)	0.3995
Cholesterol level	1.000 (0.998 to 1.002)	0.9835	1.000 (0.998 to 1.002)	0.6669
Hypertension yes vs no	0.917 (0.757 to 1.111)	0.3757	0.871 (0.700 to 1.084)	0.2153
Asthma yes vs no	1.045 (0.841 to 1.297)	0.6920	0.956 (0.748 to 1.223)	0.7230
Cirrhosis yes vs no	0.933 (0.676 to 1.287)	0.6714	0.892 (0.619 to 1.286)	0.5401
Other cancer yes vs no	0.937 (0.726 to 1.209)	0.6151	0.847 (0.634 to 1.132)	0.2622
Combined vs chemotherapy	0.932 (0.714 to 1.215)	0.6011	0.919 (0.680 to 1.241)	0.5807
Radiation vs chemotherapy	0.953 (0.733 to 1.238)	0.7160	0.794 (0.588 to 1.072)	0.1321
Surgery vs chemotherapy	0.757 (0.578 to 0.993)	0.0446	0.652 (0.478 to 0.888)	0.0067

OR, odds ratio; aOR, adjusted odds ratio; CI, confidence interval; NICRH, National Institute of Cancer Research & Hospital. Reference categories were female, Evercare Hospital Dhaka, Stage I, no family history, current smoker, no hypertension, no asthma, no cirrhosis, no other cancer history, and chemotherapy. Continuous predictors were modeled per one-unit increase.

The maximum variance inflation factor was 1.56, indicating no important multicollinearity. Joint addition of quadratic terms for age, body mass index, and cholesterol did not materially improve model fit (likelihood-ratio p = 0.609), providing no evidence of substantial departure from linearity in the logit for these continuous predictors.

### Prediction model performance and hospital-wise internal-external validation

The hospital-agnostic logistic prediction model had an apparent AUROC of 0.769 and an apparent Brier score of 0.192. When predictions were pooled across the four leave-one-hospital-out validation folds, AUROC was 0.753 and the Brier score was 0.198, indicating the expected modest reduction from apparent to out-of-hospital performance. Apparent and pooled ROC curves are shown in Fig 2.

**Fig 2:**
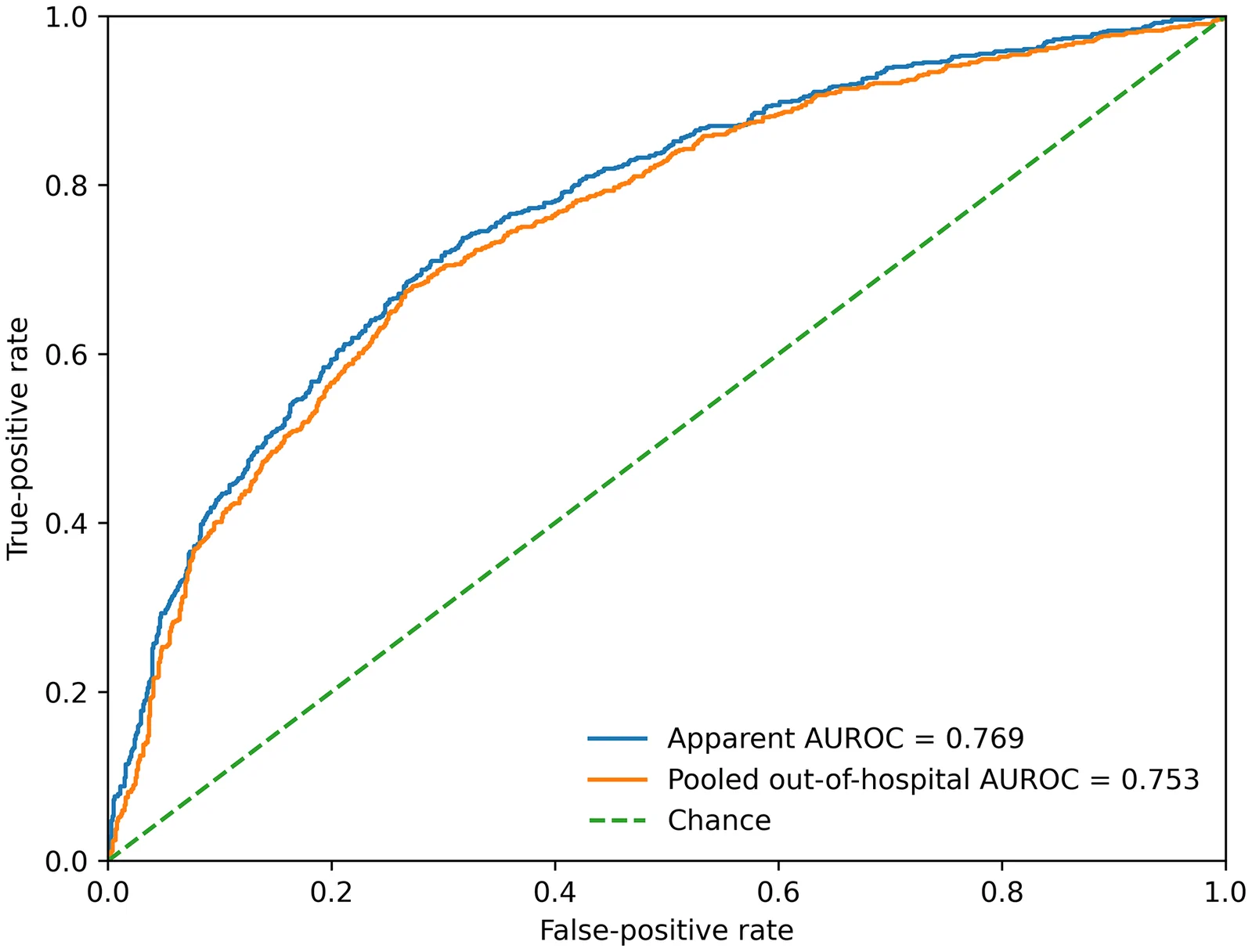
Receiver operating characteristic curves for the mortality prediction model. The figure compares apparent full-cohort discrimination with pooled leave-one-hospital-out predictions. The apparent AUROC was 0.769 and the pooled out-of-hospital AUROC was 0.753.

At the descriptive probability threshold of 0.5, pooled leave-one-hospital-out predictions yielded accuracy 0.700, precision 0.666, sensitivity/recall 0.613, and F1 score 0.638. Complete apparent, pooled, and hospital-specific validation metrics are summarized in Table 3.

**Table 3:** Apparent and leave-one-hospital-out internal-external validation performance of the hospital-agnostic prediction model.

Validation setting	N	Mortality events	AUROC	Brier score	Accuracy	Precision	Sensitivity/Recall	F1 score
Overall apparent	1782	770	0.769	0.192	0.708	0.675	0.627	0.650
Pooled LOHO	1782	770	0.753	0.198	0.700	0.666	0.613	0.638
Held-out United	443	191	0.777	0.190	0.718	0.696	0.613	0.652
Held-out NICRH	470	204	0.722	0.212	0.668	0.622	0.598	0.610
Held-out Square	436	186	0.766	0.190	0.720	0.690	0.624	0.655
Held-out Evercare	433	189	0.755	0.198	0.695	0.661	0.619	0.639

AUROC, area under the receiver operating characteristic curve. Classification metrics use a probability threshold of 0.5, which was prespecified for descriptive reporting and was not optimized.

Hospital-specific AUROCs were 0.777 for United Hospital Limited, 0.722 for NICRH, 0.766 for Square Hospital Ltd., and 0.755 for Evercare Hospital Dhaka. Thus, discrimination was moderately stable across held-out hospital sources, although this remains internal-external validation within one public dataset rather than independent external validation. Hospital-specific AUROCs are also displayed in S1 Fig.

### Calibration and decision curve analysis

Using pooled out-of-hospital predictions, the calibration intercept was −0.031 (95% CI −0.138 to 0.077) and the calibration slope was 0.911 (95% CI 0.807 to 1.015). The lowest predicted-risk decile had mean predicted mortality of 12.9% and observed mortality of 15.6%; the highest decile had mean predicted mortality of 83.0% and observed mortality of 78.8%. These findings indicate reasonable, though not perfect, calibration across hospitals (Fig 3).

**Fig 3:**
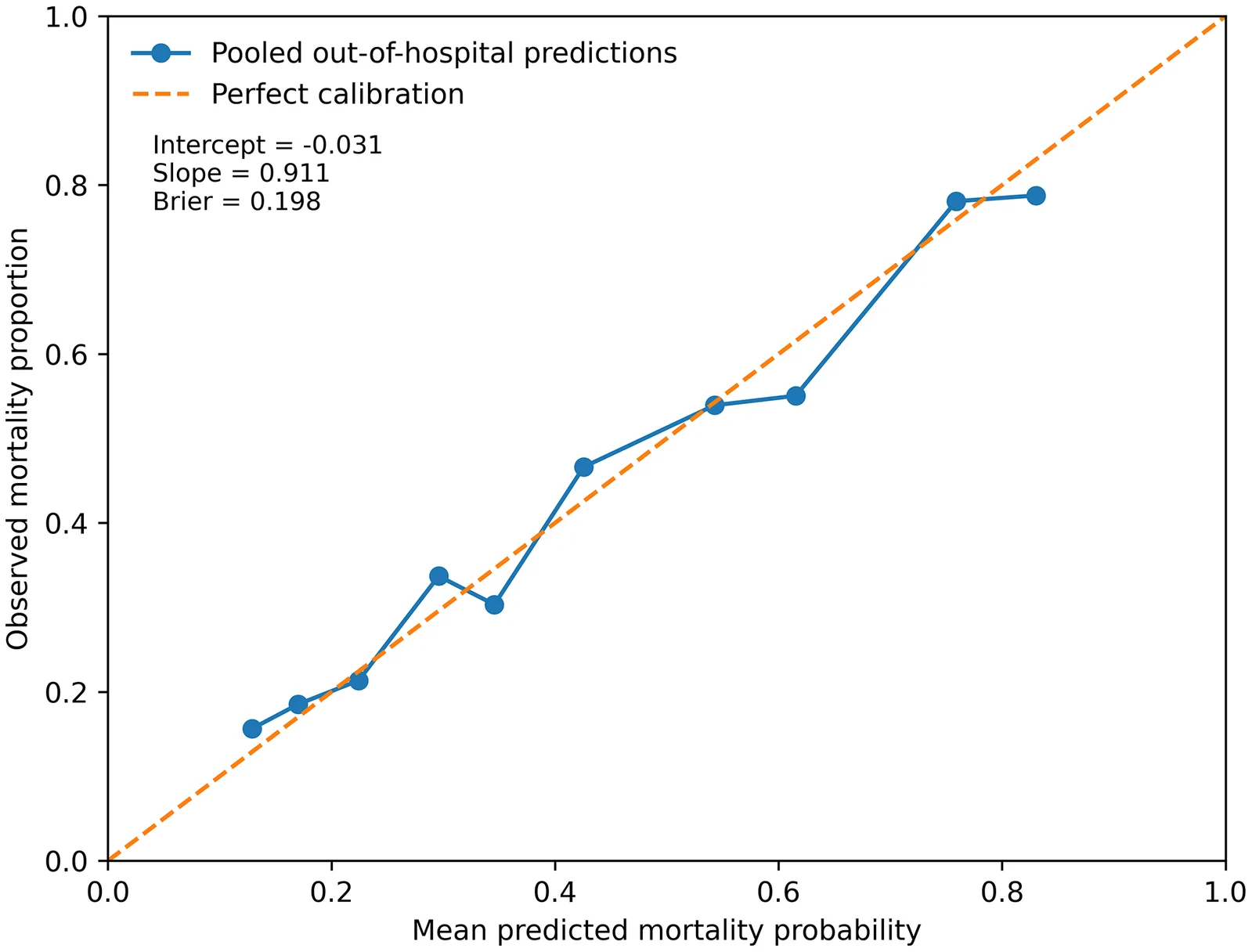
Calibration of pooled leave-one-hospital-out predictions. Mean predicted mortality probability is compared with observed mortality within predicted-risk deciles. The diagonal denotes perfect calibration. Calibration intercept, slope, and pooled Brier score are displayed within the figure.

Decision curve analysis of pooled out-of-hospital predictions showed greater net benefit for the model than both treat-all and treat-none strategies at the evaluated thresholds from approximately 0.20 to 0.75. Model net benefit was 0.2994 at threshold 0.20, 0.1895 at 0.40, and 0.0836 at 0.60 (Fig 4; S1 Table).

**Fig 4:**
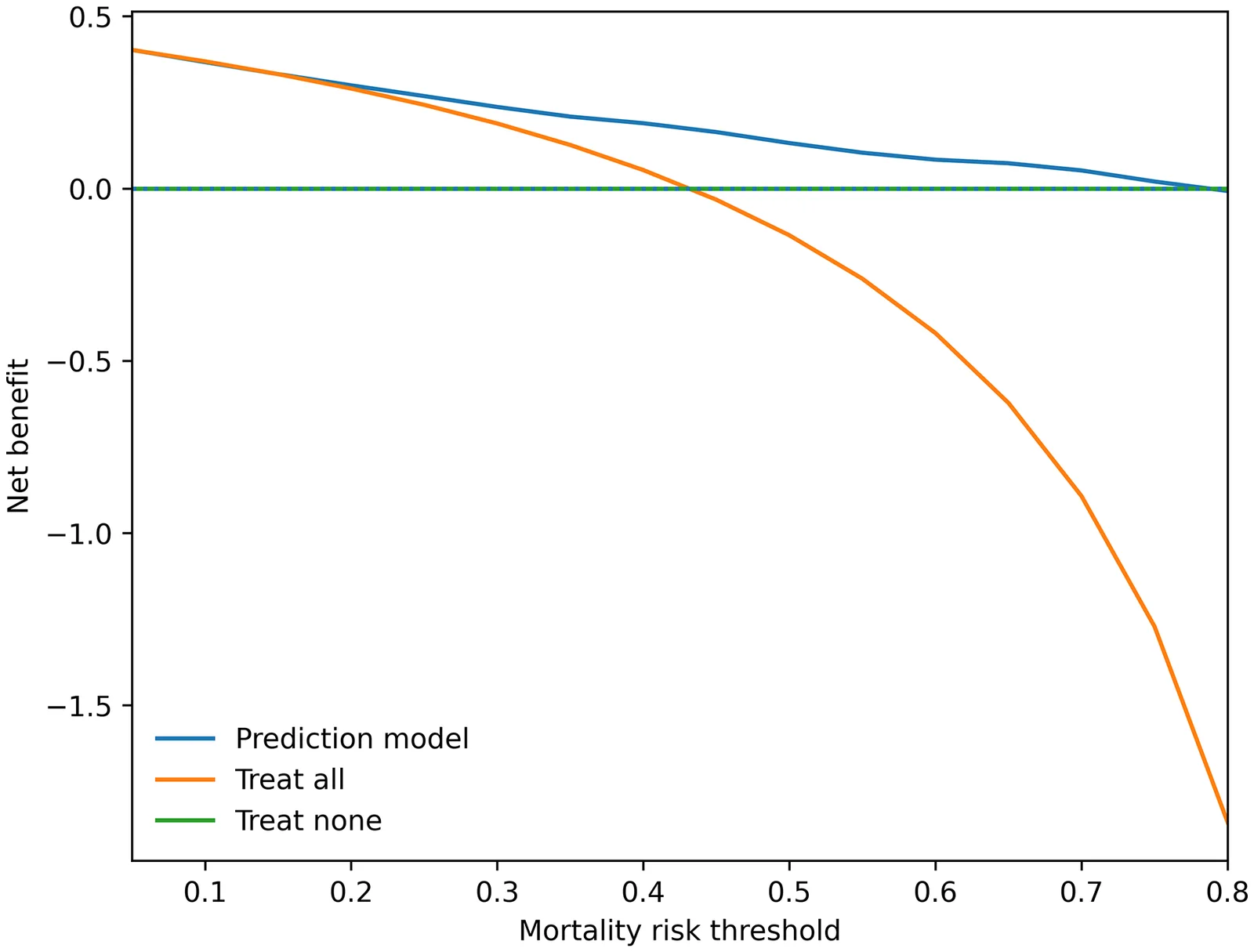
Decision curve analysis using pooled leave-one-hospital-out predictions. Net benefit of the prediction model is compared with treat-all and treat-none strategies across mortality-risk thresholds from 0.05 to 0.80.

## Discussion

### Principal findings

In this secondary analysis of 1,782 de-identified lung cancer records from four Dhaka hospitals, recorded mortality was strongly stage-related. Stage IV disease had approximately 21-fold higher adjusted odds of the recorded mortality endpoint than Stage I disease. An interpretable hospital-agnostic logistic prediction model retained moderate discrimination across leave-one-hospital-out validation, with pooled AUROC 0.753, Brier score 0.198, and calibration intercept and slope close to their ideal values.

### Interpretation of stage-related mortality

The strong stage-mortality gradient was clinically coherent with established lung cancer staging and prognosis literature [22,23]. It also reinforces the potential importance of earlier diagnosis, timely referral, and improved lung cancer care pathways in Bangladesh and comparable resource-limited settings [3–6]. However, the binary outcome in this dataset does not provide time-specific survival estimates, and the stage associations reported here are odds ratios rather than hazard ratios.

### Treatment-associated findings

Surgery was associated with lower adjusted odds of recorded mortality than chemotherapy. This association is clinically plausible because surgical candidates often have more localized disease, better functional status, and greater likelihood of receiving definitive treatment. It should not be interpreted as a causal treatment effect. The dataset does not contain important treatment-selection variables such as histologic subtype, molecular profile, ECOG performance status, surgical resectability, treatment intent, chemotherapy regimen, radiotherapy dose, or detailed treatment completion information. Residual confounding and selection bias are therefore likely.

### Hospital-wise validation and model performance

The difference between apparent AUROC (0.769) and pooled leave-one-hospital-out AUROC (0.753) was modest, and hospital-specific AUROCs ranged from 0.722 to 0.777. This suggests moderate transportability across hospital sources within Dhaka. The use of prespecified predictors, absence of data-driven feature selection or tuning, adequate events per estimated parameter, and leave-one-hospital-out refitting reduced some risks of overfitting. Nevertheless, internal-external validation does not substitute for independent external validation [24–27].

### Calibration and potential clinical utility

Calibration is essential because discrimination alone does not establish that predicted probabilities are reliable [17,18]. Pooled out-of-hospital predictions showed a calibration intercept close to zero and a slope close to one, although the lowest and highest deciles showed modest deviations between predicted and observed risk. The prediction model should therefore be considered reasonably calibrated within this dataset rather than assumed to be universally calibrated.

Decision curve analysis suggested that the model may offer greater net benefit than treat-all or treat-none strategies across an intermediate range of evaluated thresholds [19–21]. This analysis does not demonstrate improved patient outcomes or establish a clinically appropriate threshold. Prospective validation and evaluation within actual clinical workflows would be necessary before implementation.

### Strengths and limitations

Strengths of this study include use of a multi-hospital dataset, transparent reporting of the complete available cohort, explicit separation of association and prediction models, hospital-wise internal-external validation, assessment of discrimination and calibration, and decision curve analysis. The prediction model uses routinely available variables and an interpretable logistic specification.

Several limitations should be acknowledged. First, this was a retrospective secondary analysis of a public dataset, so the analysis is limited by the accuracy and preprocessing of the released data and cannot establish causality. Although the released analytic file contained no missing values, the authors could not determine whether missing-data handling or record exclusion occurred before public release. Second, the dataset does not provide a verified death date or censoring time; therefore, true overall survival, hazard ratios, and time-specific survival probabilities could not be estimated. Third, independent external validation outside the source dataset was unavailable. Fourth, important prognostic and treatment-selection variables, including histology, molecular markers, ECOG performance status, treatment intent, resectability, regimen details, radiotherapy dose, socioeconomic variables, and detailed longitudinal follow-up, were unavailable. Finally, associations with treatment may be affected by selection bias and unmeasured confounding.

## Conclusion

In this multi-hospital Bangladeshi lung cancer dataset, the recorded binary mortality endpoint was strongly associated with cancer stage. A hospital-agnostic, interpretable logistic prediction model showed moderate discrimination and reasonable calibration in leave-one-hospital-out internal-external validation and potential net benefit across an intermediate range of decision thresholds. These findings support further evaluation of routinely available clinical risk stratification in Bangladesh, while emphasizing the need for independent external and prospective validation before clinical implementation.

## Supporting information

S1 Table Calibration and decision curve analysis summary. Results are based on pooled leave-one-hospital-out predictions, including Brier score, calibration intercept and slope, predicted-risk deciles, and selected decision-curve thresholds.
(XLSX)

S2 Table Full logistic regression coefficients and model fit information. The file provides coefficients, standard errors, odds ratios, 95% confidence intervals, and p values for the univariable models and the multivariable association and prediction models.
(XLSX)

S1 Fig Leave-one-hospital-out internal-external validation AUROC by held-out hospital. The bar chart displays AUROC values when each hospital was held out and the model was fitted using the remaining three hospitals.
(TIF)

S1 Code Reproducible Python analysis code. The script performs data-quality checks, descriptive analyses, association and prediction modeling, model diagnostics, leave-one-hospital-out internal-external validation, calibration assessment, decision curve analysis, and generation of revised tables and figures.
(PY)

## Abbreviations

AUROC: area under the receiver operating characteristic curve
BMI: body mass index
CI: confidence interval
DCA: decision curve analysis
ECOG: Eastern Cooperative Oncology Group
EPV: events per estimated predictor parameter
LMIC: low- and middle-income country
NICRH: National Institute of Cancer Research & Hospital
OR: odds ratio
PM2.5: particulate matter with aerodynamic diameter <=2.5 micrometers
