# Supplementary material for: Stage-driven mortality and clinical risk stratification among lung cancer patients in Dhaka, Bangladesh: A multi-hospital secondary analysis
Source: PLoS One. 2026 Oct 15;21(10):e0359406. doi: 10.1371/journal.pone.0359406 (PMC13671521; doi:10.1371/journal.pone.0359406)
Supplement: Supplementary file 1 [file pone.0359406.s006.docx]

Response to Editor and Reviewers

**Manuscript ID: PONE-D-26-34420**

Stage-driven mortality and clinical risk stratification among lung cancer patients in Dhaka, Bangladesh: a multi-hospital secondary analysis

**Dear Dr Mitra and Reviewers,**

We thank the Academic Editor and both reviewers for their careful and constructive evaluation of our manuscript. We have revised the manuscript extensively in response to all comments. The revision clarifies the binary mortality endpoint, strengthens statistical transparency and validation, embeds the main tables, improves calibration and decision-curve reporting, standardizes terminology, corrects citation order and reference formatting, and provides reproducible analysis code. We believe these changes substantially improve the rigor, reproducibility, and clarity of the work.

Page and line references below refer to the final clean revised manuscript with continuous line numbering. Supporting Information is cited by file designation where appropriate.

# Academic Editor / Journal Requirements

**Comment:** *Please ensure that your manuscript meets PLOS ONE style requirements, including file naming and formatting requirements.*

Response: The revised manuscript has been reorganized and reformatted to conform to PLOS ONE requirements. Main tables are incorporated into the manuscript, Supporting Information is labeled separately, terminology has been standardized, and the reference list has been revised to a consistent Vancouver-style format. These changes are reflected throughout the revised manuscript.

**Comment:** *Please ensure that author-generated code underpinning the findings is shared in accordance with PLOS ONE code-sharing guidance.*

Response: We have prepared a reproducible Python analysis script and will submit it as S1 Code. The script performs the data-quality checks, descriptive analyses, univariable and multivariable logistic regression, model diagnostics, leave-one-hospital-out internal-external validation, calibration assessment, decision curve analysis, and generation of the revised tables and figures. The publicly available source dataset remains available through Mendeley Data (DOI: 10.17632/wzzxjs8gww.1). A dedicated Code Availability statement is included on page 14, lines 303-305.

**Comment:** *The ethics statement should appear only in the Methods section.*

Response: The duplicate ethics statement that previously appeared after the Data Availability section has been removed. The ethics statement now appears only in the Methods section (page 7, lines 161-165).

**Comment:** *Please include the main tables as part of the manuscript file and retain supplementary tables as Supporting Information files.*

Response: Tables 1, 2, and 3 are now embedded in the main manuscript rather than represented by spreadsheet placeholders. Table 1 begins on page 8 (line 177), Table 2 begins on page 10 (line 197), and Table 3 begins on page 11 (line 216). Supplementary Tables S1 and S2 are retained as separate Supporting Information files.

**Comment:** *The supplementary figure should be uploaded as Supporting Information, removed from the manuscript file, and its legend listed after the references.*

Response: S1 Fig is prepared as a separate Supporting Information file rather than being embedded in the manuscript body. Its legend is listed with the Supporting Information captions after the references (page 17, lines 385-387).

**Comment:** *There is no requirement to cite reviewer-suggested works unless they are relevant.*

**Response:** No reviewer-suggested citation was added solely to satisfy the review. References were retained or added only where they directly support the revised methods or interpretation.

# Reviewer 1

We appreciate the reviewer's positive assessment of the study design, stage-mortality findings, logistic-regression approach, calibration, decision-curve analysis, TRIPOD-guided reporting, and the framing of leave-one-hospital-out validation as internal-external rather than independent external validation.

**Comment:** *The absence of missing data across a large multi-site dataset is unexplained and should be addressed directly.*

Response: We agree and have clarified this point in Methods, under Study setting and population (page 5, lines 97-103). We rechecked the publicly released analytic dataset and confirmed that all 1,782 records contained complete values for the variables used in the analysis, with no duplicate patient identifiers and no duplicate complete records. Therefore, no missing-data exclusions or imputation were performed. We now explicitly state that this observation refers to the public dataset as released to us and that, as secondary analysts, we cannot determine whether data cleaning, missing-data handling, or record exclusion occurred before public release. This distinction avoids implying that the original hospital records necessarily contained no missing information.

**Comment:** *Statistical transparency is incomplete because there is no Events Per Variable statement and confidence intervals are missing from in-text odds-ratio statements.*

Response: We have added an events-per-estimated-parameter statement in Methods (page 6, lines 124-140) and 95% confidence intervals to the principal in-text odds-ratio statements in the Abstract (page 2, lines 31-40) and Results (page 10, lines 190-196). The multivariable association model contains 13 predictor variables corresponding to 21 non-intercept coefficients after categorical expansion. With 770 recorded mortality events, this yields 36.7 events per estimated predictor parameter. The hospital-agnostic prediction model contains 18 non-intercept coefficients, corresponding to 42.8 events per estimated predictor parameter. The revised text reports Stage II 2.54 (95% CI 1.86 to 3.46), Stage III 6.66 (95% CI 4.86 to 9.14), Stage IV 20.77 (95% CI 14.46 to 29.83), and surgery versus chemotherapy 0.65 (95% CI 0.48 to 0.89).

**Comment:** *Tables 1 to 3 were not visible in the manuscript and were represented only by placeholders, preventing full technical and statistical review.*

Response: We apologize for this submission-formatting problem. Tables 1, 2, and 3 are now fully embedded in the main manuscript (pages 8-11). Table 1 presents baseline characteristics by recorded outcome status, Table 2 presents univariable and multivariable logistic-regression results with odds ratios, 95% confidence intervals, and P values, and Table 3 presents apparent and leave-one-hospital-out internal-external validation metrics. We additionally provide S2 Table with full model coefficients, standard errors, confidence intervals, P values, and model-fit information to improve reproducibility and statistical transparency.

# Reviewer 2

**Comment:** *Please clarify the distinction between mortality status and survival analysis. The available follow-up interval is not necessarily true overall survival time. Explain why logistic regression was selected rather than a time-to-event approach and clarify the implications of not having a reliable survival time variable.*

Response: We agree and have substantially revised the manuscript to make this distinction explicit. The primary endpoint is consistently described as a recorded binary mortality status derived from the source variable survived. Methods now states that the dataset does not provide a verified death date or suitable censoring time, so diagnosis and end-treatment dates are not used for Kaplan-Meier, Cox proportional-hazards, or other time-to-event analyses (page 5, lines 104-115). Logistic regression was therefore selected because the available outcome is binary rather than a valid time-to-event endpoint. The manuscript explicitly states that the reported odds ratios and predicted probabilities should not be interpreted as hazard ratios, median overall survival, or time-specific survival probabilities. The same distinction is reiterated in the Discussion (page 12, lines 247-250), and survival has been removed as a manuscript keyword.

**Comment:** *The manuscript uses multiple machine-learning approaches for prediction/model development. Please provide details regarding feature selection, model training, parameter optimization, cross-validation, independent validation, and prevention of overfitting.*

Response: Thank you for raising this point. We apologize that the original wording may have created the impression that multiple machine-learning algorithms were evaluated. The study uses logistic regression throughout and does not compare multiple machine-learning models. The revised Methods distinguishes (1) a multivariable association model that includes hospital source and is used for adjusted odds ratios and (2) a hospital-agnostic logistic prediction model that excludes hospital source a priori so that an entire hospital can be held out for validation (page 6, lines 124-150). Predictors were prespecified from variables available in the source dataset. No automated feature selection, stepwise selection, hyperparameter optimization, or data-driven probability-threshold optimization was performed (page 6, lines 137-140). The 0.5 threshold is reported only for descriptive classification metrics (page 7, lines 151-153). All leave-one-hospital-out folds use the same unpenalized logistic-regression specification (page 7, lines 144-150). Potential overfitting was addressed through adequate events per estimated parameter, absence of data-driven feature selection or tuning, and leave-one-hospital-out internal-external validation. Pooled out-of-hospital predictions yielded AUROC 0.753 and Brier score 0.198 compared with apparent AUROC 0.769 and Brier score 0.192 (page 10, lines 205-215). Calibration based on pooled out-of-hospital predictions produced an intercept of -0.031 (95% CI -0.138 to 0.077) and slope of 0.911 (95% CI 0.807 to 1.015) (page 11, lines 224-232). We continue to state clearly that no independent external validation dataset was available.

**Comment:** *Carefully check the numbering and placement of citations. All in-text citations should appear sequentially and correspond correctly to the reference list.*

Response: We have rebuilt the citation numbering according to first appearance in the manuscript. In-text citations are now sequential and correspond to the revised reference list (pages 15-17).

**Comment:** *The reference list should be checked for consistency with PLOS ONE reference style.*

Response: The complete reference list has been reviewed and reformatted to a consistent Vancouver-style presentation appropriate for PLOS ONE, including standardized author presentation, journal information, volume and page or article numbers where available, and DOI information where available (pages 15-17).

**Comment:** *Terms such as survival, mortality, and follow-up should be used consistently and precisely because the calculated interval does not represent true overall survival.*

Response: We agree. Terminology has been revised throughout. The manuscript now uses recorded mortality status or recorded binary mortality endpoint for the modeled outcome. The date variables are no longer used as a survival or follow-up endpoint, survival has been removed as a manuscript keyword, and Methods explicitly states that no time-to-event quantities were estimated (page 5, lines 104-115).

**Comment:** *The manuscript would benefit from careful English-language editing to improve sentence structure, clarity, and consistency of terminology.*

Response: The manuscript has undergone a full language and consistency edit. Sentence structure has been simplified where needed, terminology has been standardized, redundant wording has been removed, and statistical statements have been revised for precision throughout the manuscript.

# Additional statistical revisions made to strengthen the manuscript

- The primary prediction analysis now uses pooled leave-one-hospital-out predictions to estimate out-of-hospital discrimination, prediction error, calibration, and decision-curve performance.
- Calibration is now reported with Brier score, calibration intercept, calibration slope, and predicted-risk deciles rather than relying only on apparent full-cohort calibration.
- Decision curve analysis is now based on pooled out-of-hospital predictions. At the evaluated 0.05 increments, the model had greater net benefit than treat-all and treat-none strategies from approximately 0.20 to 0.75, rather than claiming superiority across the entire 0.05 to 0.80 range.
- Multicollinearity was assessed using variance inflation factors, with all VIFs below approximately 1.6. Potential nonlinearity of age, BMI, and cholesterol was examined by adding quadratic terms jointly; the likelihood-ratio test did not support meaningful improvement in fit (P = 0.609).
- The revised Supporting Information includes S1 Table for calibration and decision-curve results, S2 Table for full regression coefficients and model-fit information, S1 Figure for hospital-specific internal-external validation AUROCs, and S1 Code for reproducible analysis.

Additional transparency: In accordance with PLOS policy, the revised Methods includes an explicit disclosure of the use of OpenAI ChatGPT (GPT-5.6) during revision for language editing, manuscript organization, and assistance with Python code development and reproducibility checks. The disclosure states that all AI-assisted text, code, statistical outputs, references, tables, and figures were reviewed and verified by the authors, who retain full responsibility for the final work (page 8, lines 166-170).

*We again thank the Academic Editor and Reviewers for the constructive comments, which led to substantial improvements in the statistical transparency, validation framework, terminology, reproducibility, and presentation of the manuscript.*
