# Supplementary material for: Stage-driven mortality and clinical risk stratification among lung cancer patients in Dhaka, Bangladesh: A multi-hospital secondary analysis
Source: PLoS One. 2026 Oct 15;21(10):e0359406. doi: 10.1371/journal.pone.0359406 (PMC13671521; doi:10.1371/journal.pone.0359406)
Supplement: Peer review history

# Peer review: Decision Letter 0

**DOI:** 10.1371/journal.pone.0359406.r001

**Authors:** Satabdi Mitra

10 Aug 2026

Dear Dr. Das,

Thank you for submitting your manuscript to PLOS ONE. After careful consideration, we feel that it has merit but does not fully meet PLOS ONE’s publication criteria as it currently stands. Therefore, we invite you to submit a revised version of the manuscript that addresses the points raised during the review process.

Please submit your revised manuscript by Oct 09 2026 11:59PM. If you will need more time than this to complete your revisions, please reply to this message or contact the journal office at plosone@plos.org. When you're ready to submit your revision, log on to https://www.editorialmanager.com/pone/ and select the 'Submissions Needing Revision' folder to locate your manuscript file.

- A letter that responds to each point raised by the academic editor and reviewer(s). You should upload this letter as a separate file labeled 'Response to Reviewers'.
- A marked-up copy of your manuscript that highlights changes made to the original version. You should upload this as a separate file labeled 'Revised Manuscript with Track Changes'.
- An unmarked version of your revised paper without tracked changes. You should upload this as a separate file labeled 'Manuscript'.

If you would like to make changes to your financial disclosure, please include your updated statement in your cover letter. Guidelines for resubmitting your figure files are available below the reviewer comments at the end of this letter.

If applicable, we recommend that you deposit your laboratory protocols in protocols.io to enhance the reproducibility of your results. Protocols.io assigns your protocol its own identifier (DOI) so that it can be cited independently in the future. For instructions see: https://journals.plos.org/plosone/s/submission-guidelines#loc-laboratory-protocols. Additionally, PLOS ONE offers an option for publishing peer-reviewed Lab Protocol articles, which describe protocols hosted on protocols.io. Read more information on sharing protocols at https://plos.org/protocols?utm_medium=editorial-email&utm_source=authorletters&utm_campaign=protocols.

As the corresponding author, your ORCID iD is verified in the submission system and will appear in the published article. PLOS supports the use of ORCID, and we encourage all coauthors to register for an ORCID iD and use it as well. Please encourage your coauthors to verify their ORCID iD within the submission system before final acceptance, as unverified ORCID iDs will not appear in the published article. *Only*  the individual author can complete the verification step; PLOS staff *cannot*  verify ORCID iDs on behalf of authors.

We look forward to receiving your revised manuscript.

Kind regards,

Satabdi Mitra, M.D(Community Medicine )

Academic Editor

PLOS One

Journal Requirements:

When submitting your revision, we need you to address these additional requirements.

1. Please ensure that your manuscript meets PLOS ONE's style requirements, including those for file naming. The PLOS ONE style templates can be found at

https://journals.plos.org/plosone/s/file?id=wjVg/PLOSOne_formatting_sample_main_body.pdf and

https://journals.plos.org/plosone/s/file?id=ba62/PLOSOne_formatting_sample_title_authors_affiliations.pdf

2. Please note that PLOS One has specific guidelines on code sharing for submissions in which author-generated code underpins the findings in the manuscript. In these cases, we expect all author-generated code to be made available without restrictions upon publication of the work. Please review our guidelines at https://journals.plos.org/plosone/s/materials-and-software-sharing#loc-sharing-code and ensure that your code is shared in a way that follows best practice and facilitates reproducibility and reuse.

3. Your ethics statement should only appear in the Methods section of your manuscript. If your ethics statement is written in any section besides the Methods, please delete it from any other section.

4. Please include your tables as part of your main manuscript and remove the individual files. Please note that supplementary tables (should remain/ be uploaded) as separate ""supporting information"" files"""

5. We notice that your supplementary figure is uploaded with the file type 'Figure'. Please amend the file type to 'Supporting Information'. Please ensure that each Supporting Information file has a legend listed in the manuscript after the references list.

6. We notice that your supplementary figure is included in the manuscript file. Please remove them and upload them with the file type 'Supporting Information'. Please ensure that each Supporting Information file has a legend listed in the manuscript after the references list.

7. If the reviewer comments include a recommendation to cite specific previously published works, please review and evaluate these publications to determine whether they are relevant and should be cited. There is no requirement to cite these works unless the editor has indicated otherwise.

[Note: HTML markup is below. Please do not edit.]

Reviewers' comments:

Reviewer's Responses to Questions

**Comments to the Author**

1. Is the manuscript technically sound, and do the data support the conclusions?

Reviewer #1: Partly

Reviewer #2: Partly

**********

2. Has the statistical analysis been performed appropriately and rigorously?

Reviewer #1: No

Reviewer #2: Yes

**********

3. Have the authors made all data underlying the findings in their manuscript fully available?

The PLOS Data policy

Reviewer #1: Yes

Reviewer #2: Yes

**********

4. Is the manuscript presented in an intelligible fashion and written in standard English?

Reviewer #1: Yes

Reviewer #2: Yes

**********

Reviewer #1: This is a reasonably well-conducted secondary analysis of a four-hospital lung cancer dataset from Dhaka, Bangladesh, demonstrating a strong, clinically expected stage-mortality gradient and a logistic-regression mortality model with acceptable discrimination (AUROC 0.769), good calibration, and positive net benefit on decision curve analysis. The authors appropriately use TRIPOD-guided reporting and correctly frame their leave-one-hospital-out validation as internal-external rather than true external validation. The main concerns are: (1) an unexplained absence of missing data across a large, multi-site dataset, which should be addressed directly; (2) incomplete statistical transparency (no Events Per-Variable (EPV) statement, CIs missing from in-text OR statements); (3) tables not visible/embedded in the manuscript file provided for review;

Table 1 (baseline characteristics), Table 2 (regression results with ORs and CIs), and Table 3 (model performance/validation) are referenced only as "[Insert TableX_final.xlsx here]" placeholders in the manuscript text provided for review. A full technical/statistical review cannot be completed without seeing the actual table contents (e.g., verifying CIs, checking for any inconsistency with text). Suggested improvement

Reviewer #2: 1. The authors should clarify the distinction between mortality status and survival analysis in this study. Since the available follow-up interval is not necessarily the true overall survival time, the term “survival analysis” should be used with caution. Kindly explain why logistic regression for mortality was selected rather than a time-to-event approach, and clarify the implications of not having a reliable survival time variable.

2. The manuscript uses multiple machine-learning approaches for prediction/model development. Kindly provide additional details regarding feature selection, model training, parameter optimization, cross-validation, and independent validation. The authors should also clarify how potential overfitting was minimized.

3. Carefully check the numbering and placement of citations throughout the manuscript. All in-text citations should appear sequentially and consistently and should correspond correctly to the reference list.

4. The reference list should be carefully checked for consistency with the PLOS ONE reference style.

5. The manuscript uses terms such as “survival,” “mortality,” and “follow-up.” Because the authors acknowledge that the calculated interval does not represent true overall survival, these terms should be used consistently and precisely.

6. The manuscript would benefit from careful English language editing to improve sentence structure, clarity, and consistency of terminology.

**********

what does this mean?). If published, this will include your full peer review and any attached files.

If you choose “no”, your identity will remain anonymous but your review may still be made public.

**Do you want your identity to be public for this peer review?**  For information about this choice, including consent withdrawal, please see our Privacy Policy

Reviewer #1: No

Reviewer #2: **Yes:**  Dr Ramya N

**********

[NOTE: If reviewer comments were submitted as an attachment file, they will be attached to this email and accessible via the submission site. Please log into your account, locate the manuscript record, and check for the action link "View Attachments". If this link does not appear, there are no attachment files.]

To ensure your figures meet our technical requirements, please review our figure guidelines: https://journals.plos.org/plosone/s/figures

You may also use PLOS’s free figure tool, NAAS, to help you prepare publication quality figures: https://journals.plos.org/plosone/s/figures#loc-tools-for-figure-preparation.

NAAS will assess whether your figures meet our technical requirements by comparing each figure against our figure specifications.

# Author comment: Author response to Decision Letter 1

**DOI:** 10.1371/journal.pone.0359406.r002

9 Sep 2026

Thank you to the Academic Editor and both reviewers for their careful and constructive assessment of our manuscript. We have addressed all editorial and reviewer comments in the revised manuscript. A detailed point-by-point response, including the changes made for each comment, has been uploaded as the file “Response to Reviewers.” We have also provided a marked revised manuscript, updated reporting checklists, revised supporting information, and reproducible analysis code.

Attachment Submitted filename: PLOS_ONE_Response_to_Reviewers_FINAL.docx

# Editor report: Decision Letter 1

**DOI:** 10.1371/journal.pone.0359406.r003

**Authors:** Satabdi Mitra

15 Sep 2026

Stage-driven mortality and clinical risk stratification among lung cancer patients in Dhaka, Bangladesh: a multi-hospital secondary analysis

PONE-D-26-34420R1

Dear Dr. Sourov Das,

We’re pleased to inform you that your manuscript has been judged scientifically suitable for publication and will be formally accepted for publication once it meets all outstanding technical requirements.

Within one week, you’ll receive an e-mail detailing the required amendments. When these have been addressed, you’ll receive a formal acceptance letter and your manuscript will be scheduled for publication.

An invoice will be generated when your article is formally accepted. Please note, if your institution has a publishing partnership with PLOS and your article meets the relevant criteria, all or part of your publication costs will be covered. Please make sure your user information is up-to-date by logging into Editorial Manager at Editorial Manager® and clicking the ‘Update My Information' link at the top of the page. For questions related to billing, please contact billing support.

If your institution or institutions have a press office, please notify them about your upcoming paper to help maximize its impact. If they’ll be preparing press materials, please inform our press team as soon as possible -- no later than 48 hours after receiving the formal acceptance. Your manuscript will remain under strict press embargo until 2 pm Eastern Time on the date of publication. For more information, please contact onepress@plos.org.

Kind regards,

Satabdi Mitra, M.D(Community Medicine )

Academic Editor

PLOS One

Additional Editor Comments (optional):

Reviewers' comments:

# Editor report: Acceptance letter

**DOI:** 10.1371/journal.pone.0359406.r004

**Authors:** Satabdi Mitra

PONE-D-26-34420R1

PLOS One

Dear Dr. Das,

I'm pleased to inform you that your manuscript has been deemed suitable for publication in PLOS One. Congratulations! Your manuscript is now being handed over to our production team.

At this stage, our production department will prepare your paper for publication. This includes ensuring the following:

* All references, tables, and figures are properly cited

* All relevant supporting information is included in the manuscript submission,

* There are no issues that prevent the paper from being properly typeset

You will receive further instructions from the production team, including instructions on how to review your proof when it is ready. Please keep in mind that we are working through a large volume of accepted articles, so please give us a few days to review your paper and let you know the next and final steps.

Lastly, if your institution or institutions have a press office, please let them know about your upcoming paper now to help maximize its impact. If they'll be preparing press materials, please inform our press team within the next 48 hours. Your manuscript will remain under strict press embargo until 2 pm Eastern Time on the date of publication. For more information, please contact onepress@plos.org.

You will receive an invoice from PLOS for your publication fee after your manuscript has reached the completed accept phase. If you receive an email requesting payment before acceptance or for any other service, this may be a phishing scheme. Learn how to identify phishing emails and protect your accounts at https://explore.plos.org/phishing.

If we can help with anything else, please email us at customercare@plos.org.

Thank you for submitting your work to PLOS One and supporting open access.

Kind regards,

PLOS One Editorial Office Staff

on behalf of

Dr Satabdi Mitra

Academic Editor

PLOS One
